# Supplementary figures and images for: Understanding the Genome-Wide Transcription Response To Various cAMP Levels in Bacteria Using Phenomenological Models
Source: mSystems. 2022 Nov 21;7(6):e00900-22. doi: 10.1128/msystems.00900-22 (PMC9765429; doi:10.1128/msystems.00900-22)

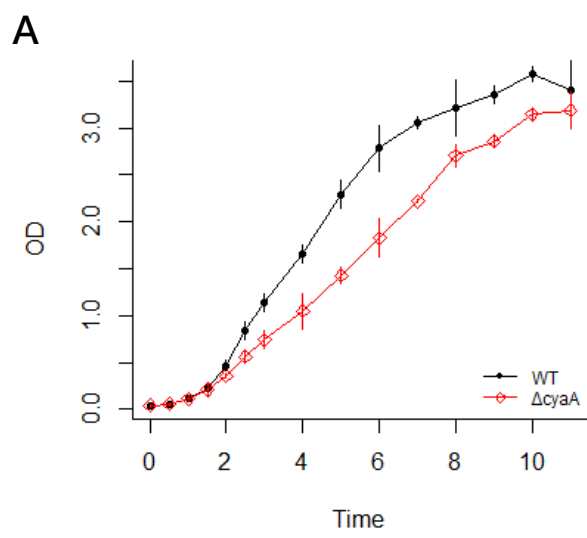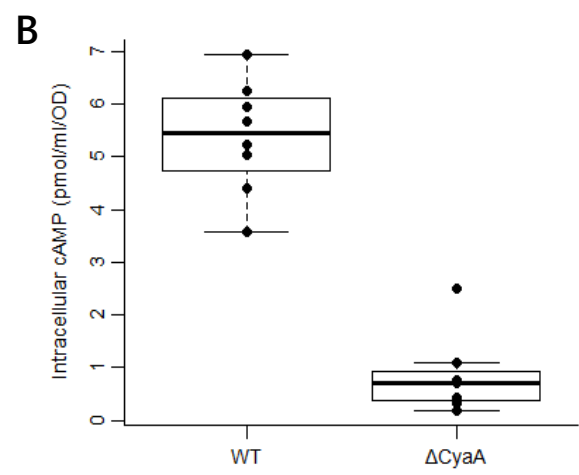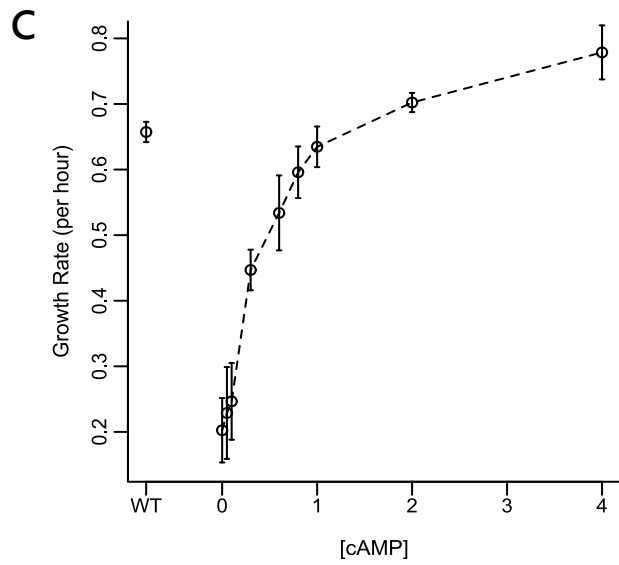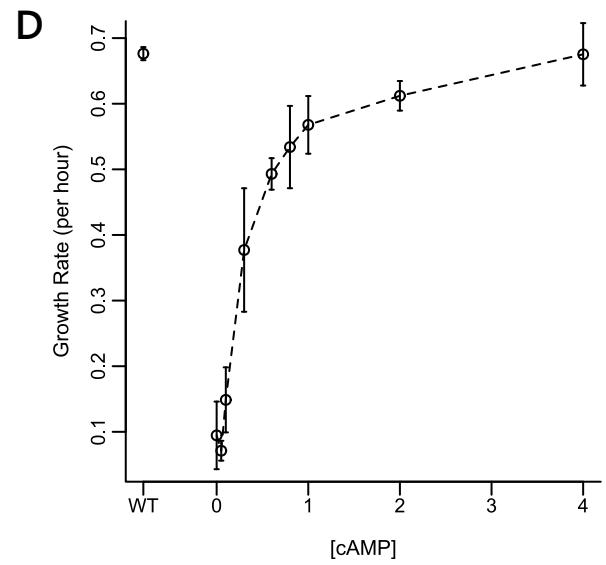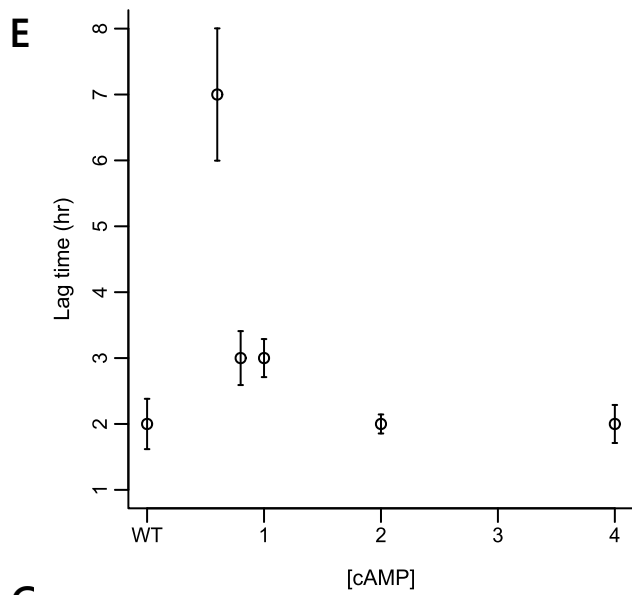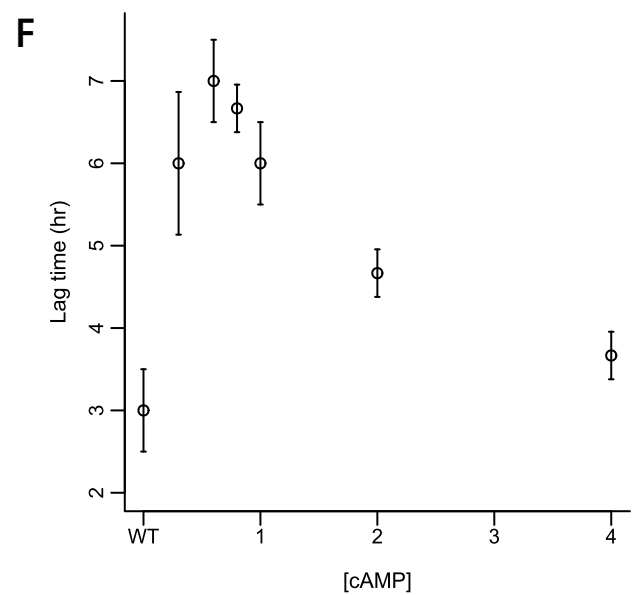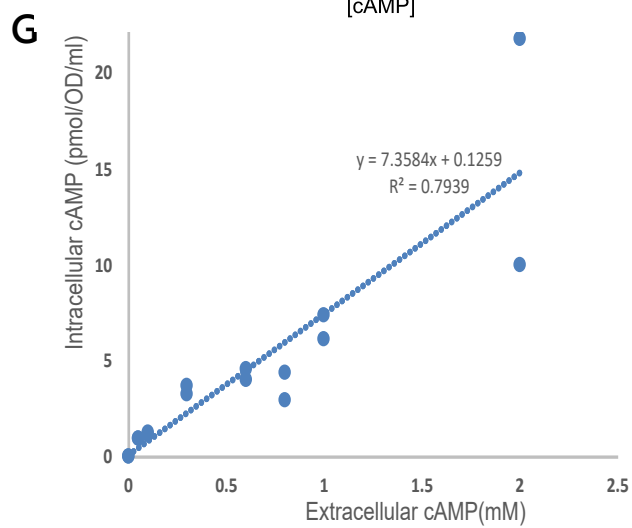

Supplement: FIG S1 [file msystems.00900-22-s0002.pdf]

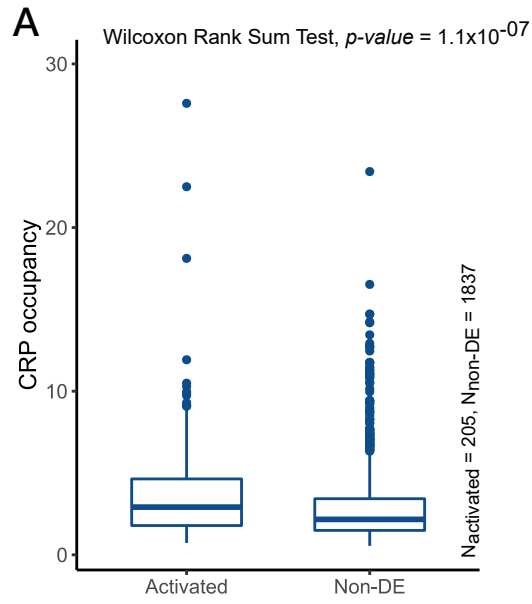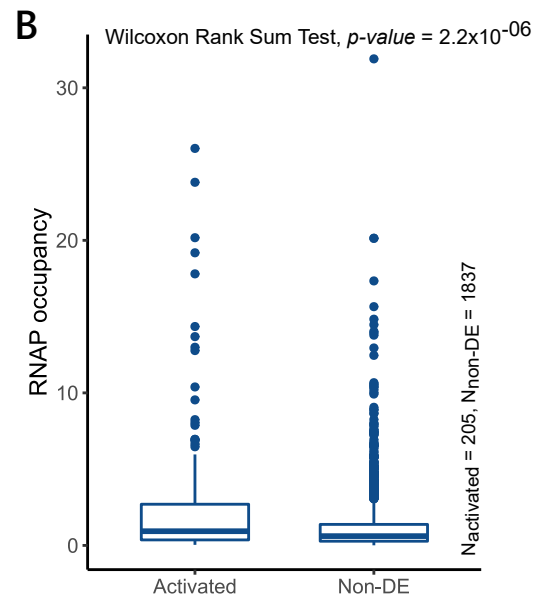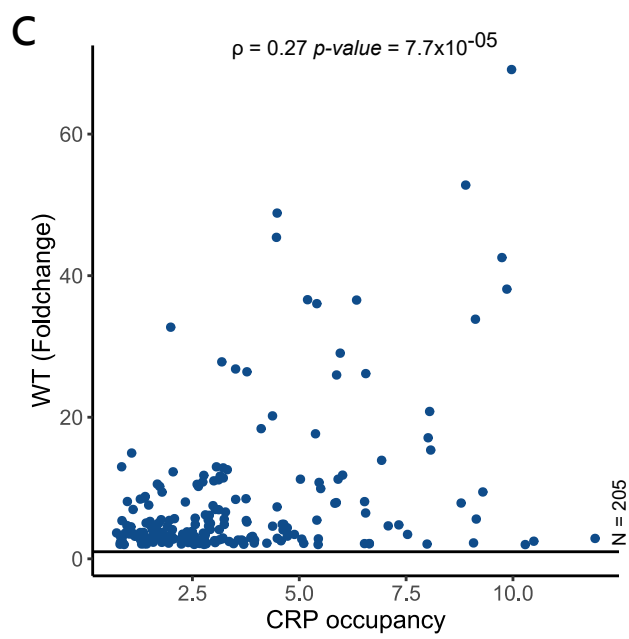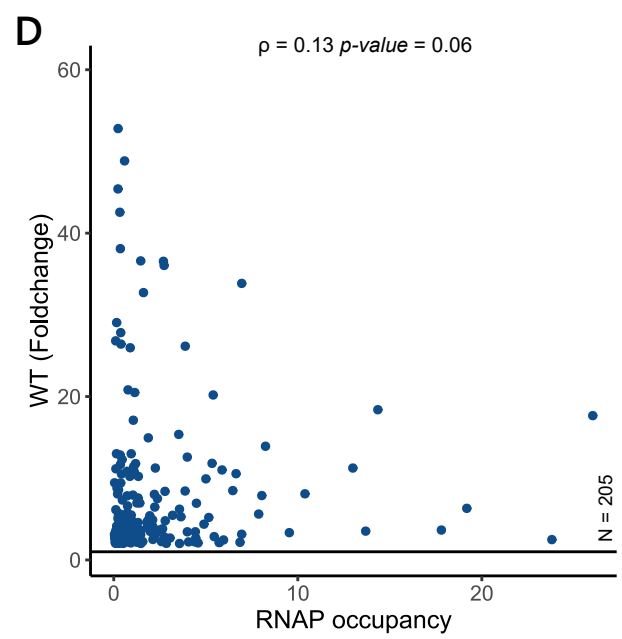

Supplement: FIG S3 [file msystems.00900-22-s0004.pdf]

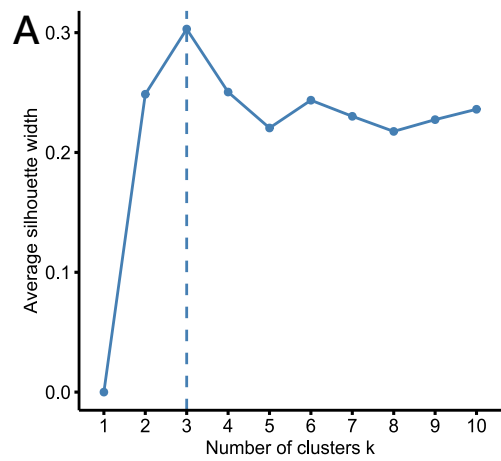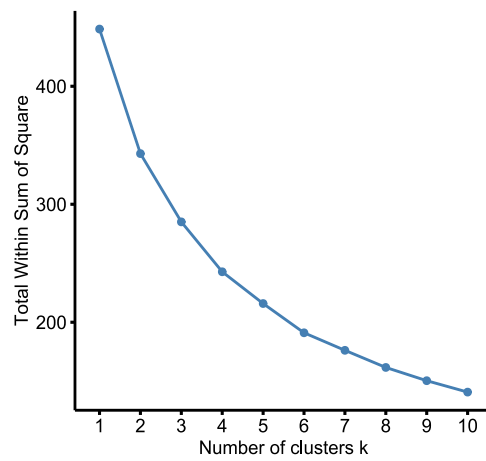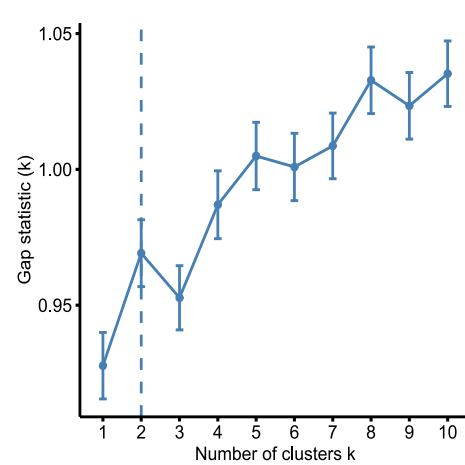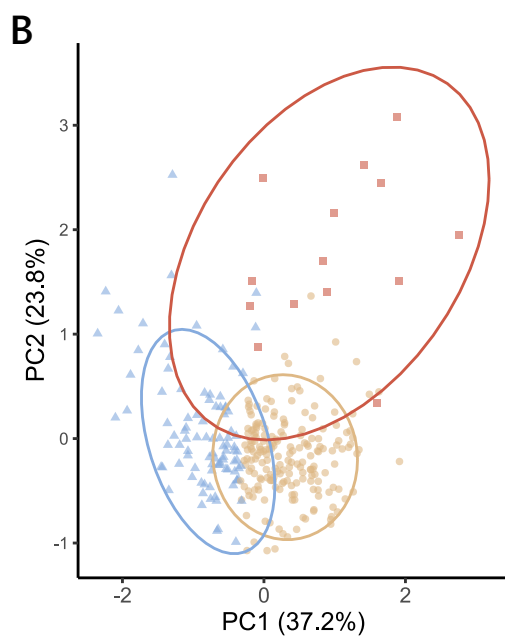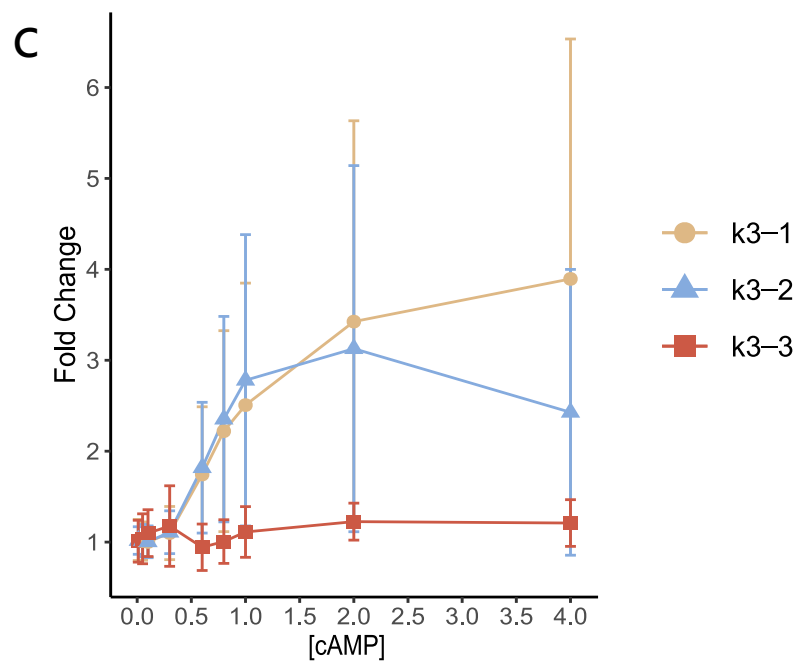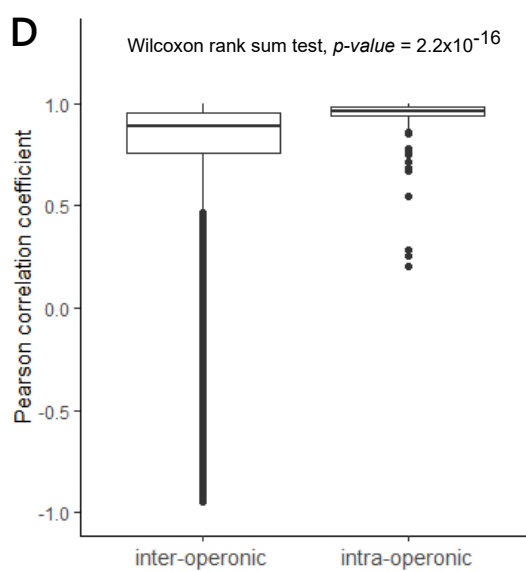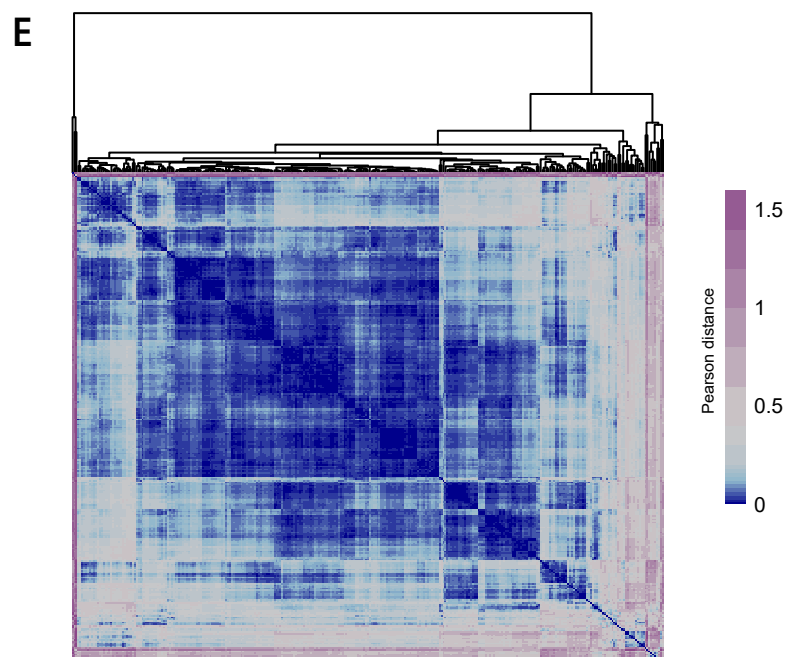

Supplement: FIG S4 [file msystems.00900-22-s0005.pdf]

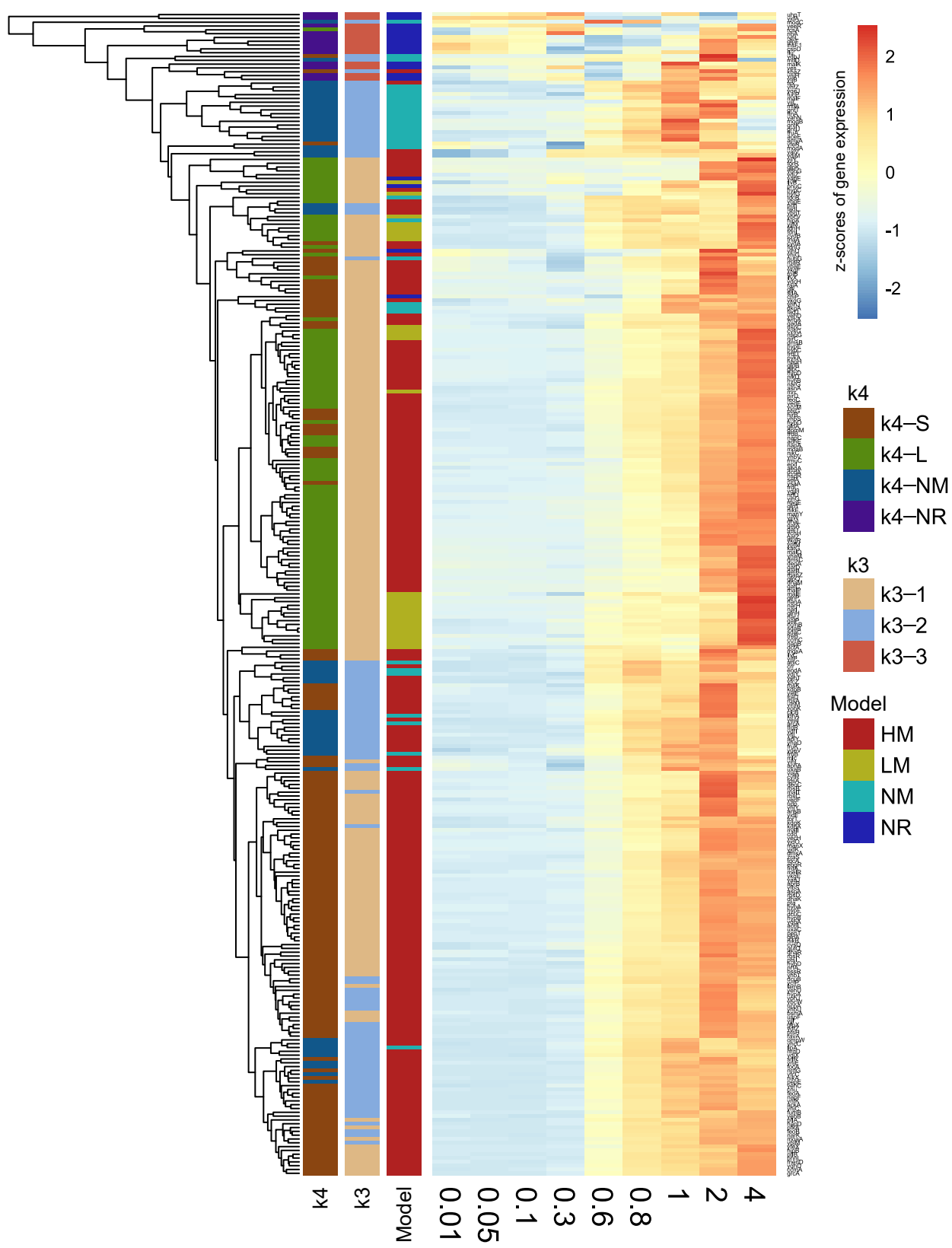

Supplement: FIG S5 [file msystems.00900-22-s0006.pdf]

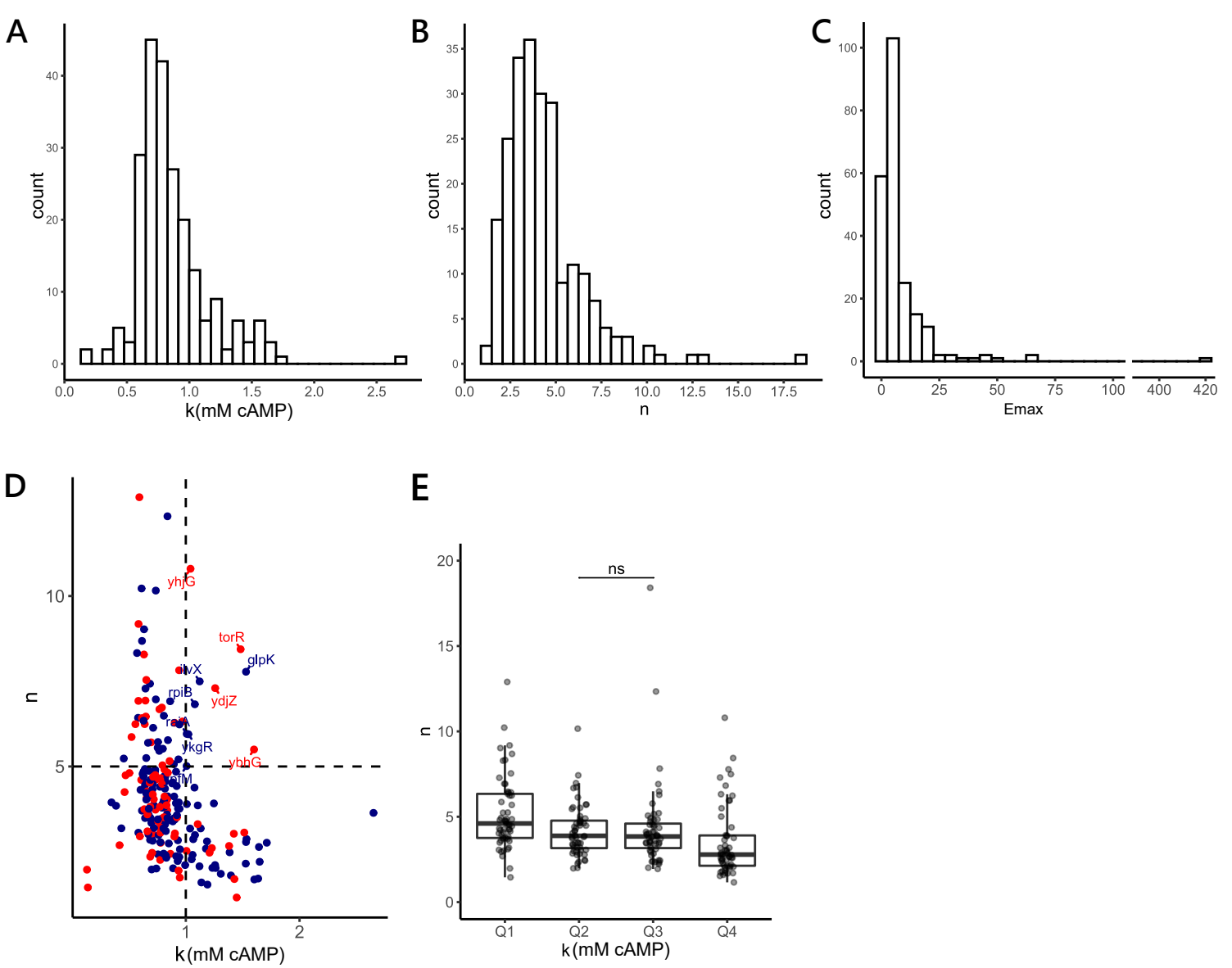

Supplement: FIG S6 [file msystems.00900-22-s0007.pdf]

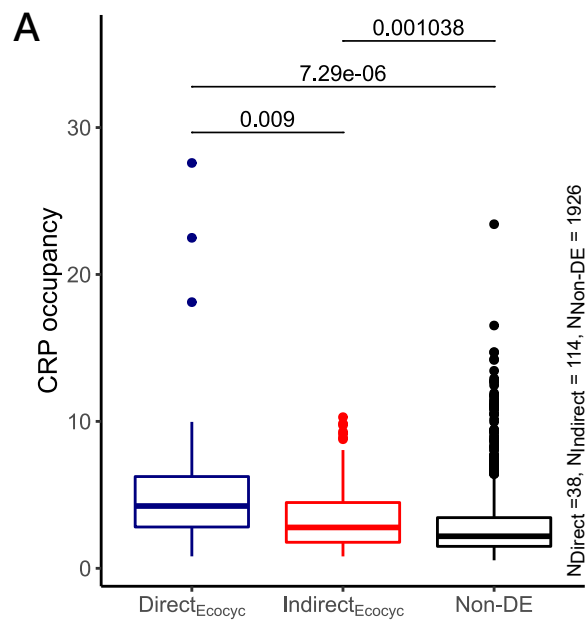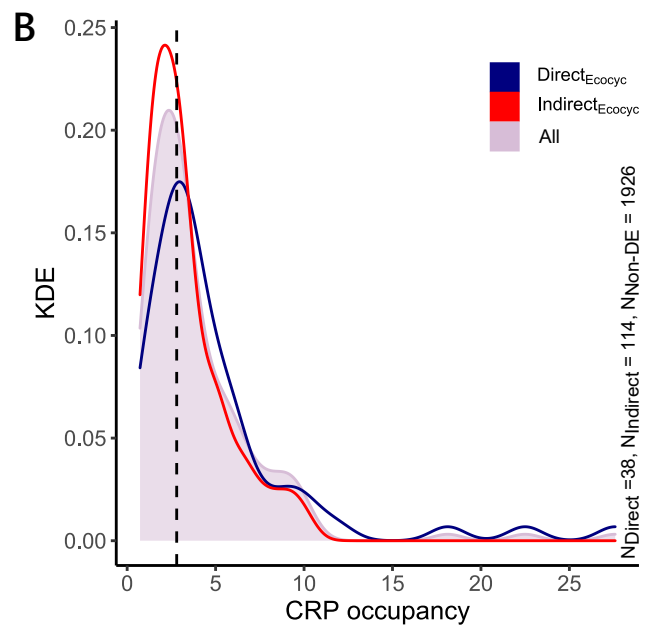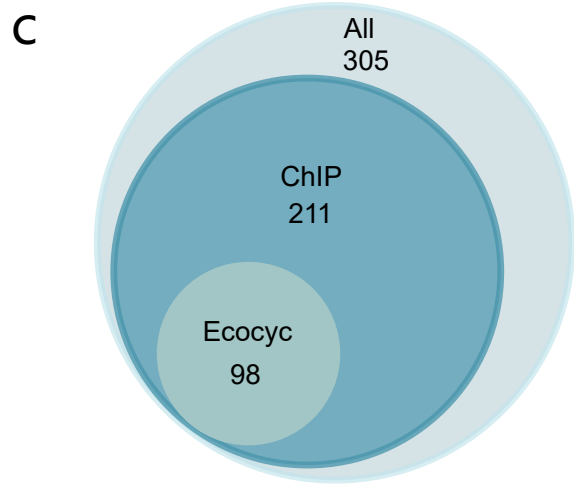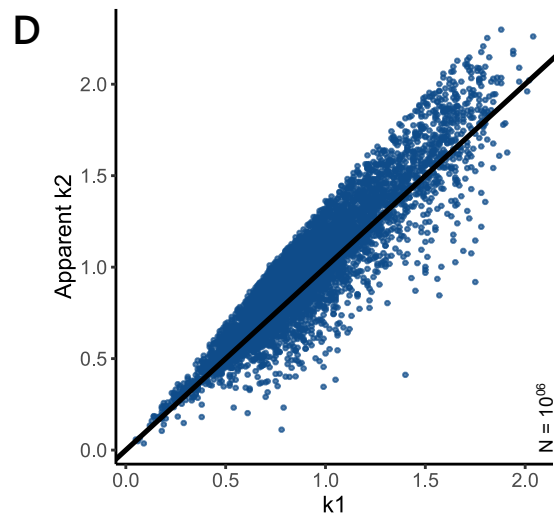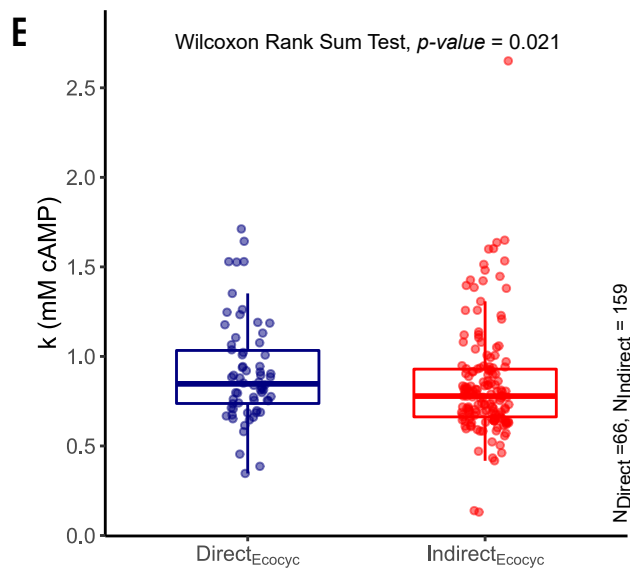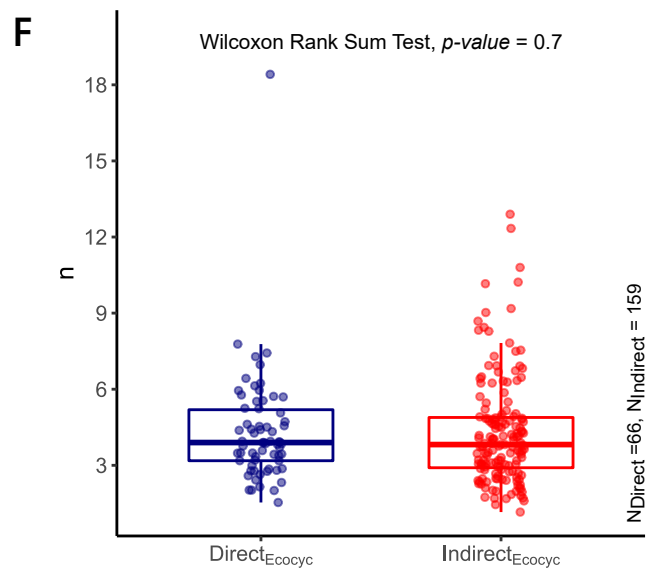

Supplement: FIG S7 [file msystems.00900-22-s0008.pdf]

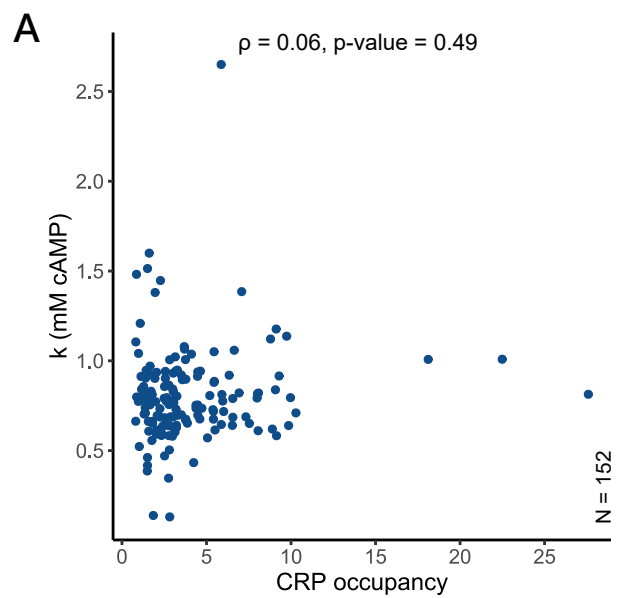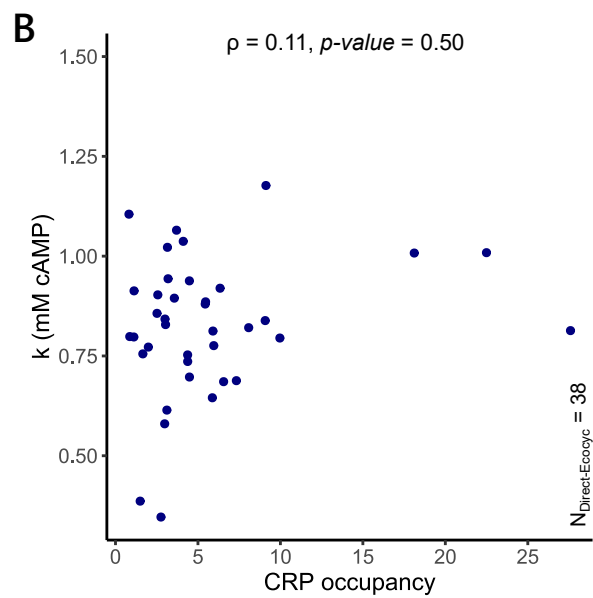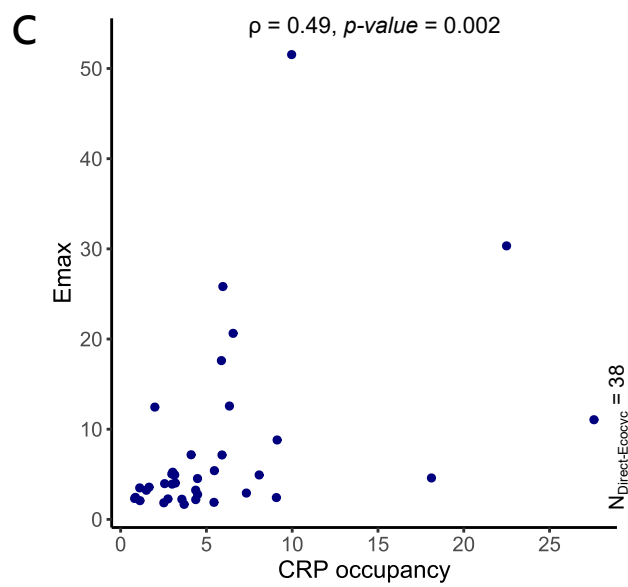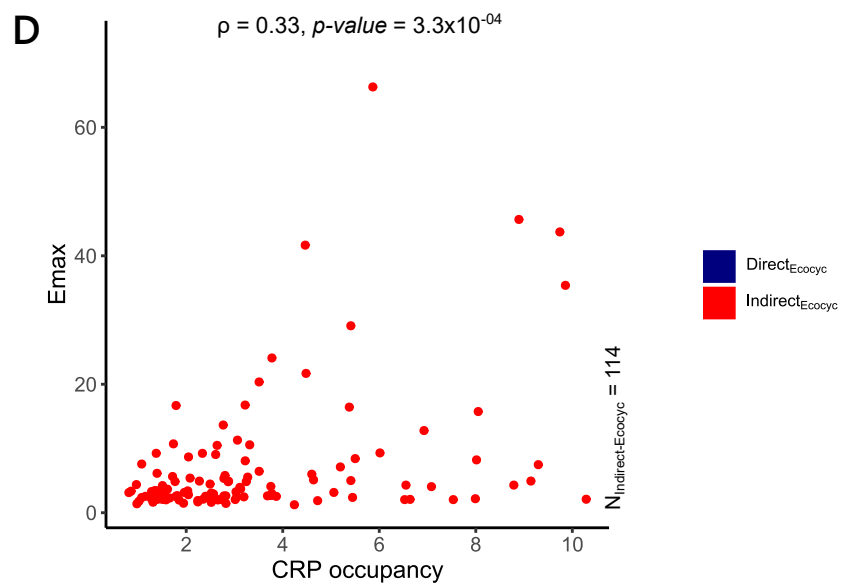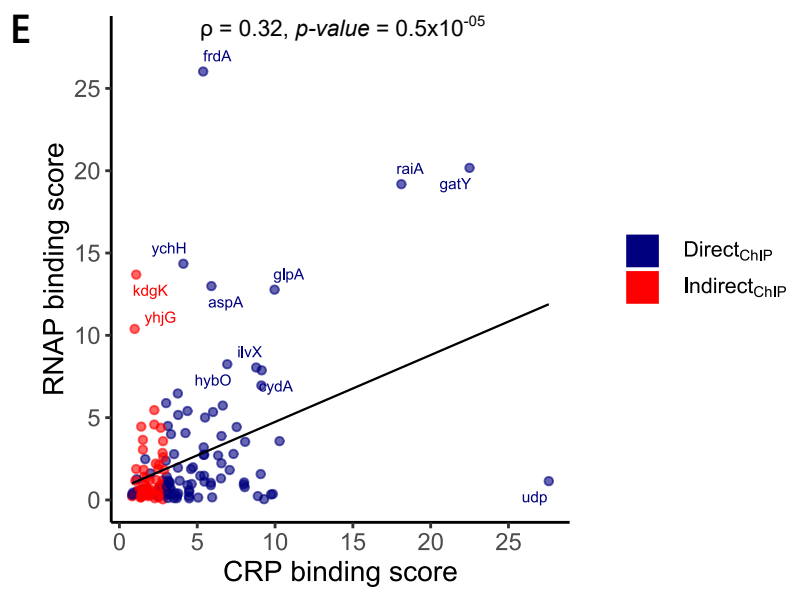

Supplement: FIG S8 [file msystems.00900-22-s0009.pdf]

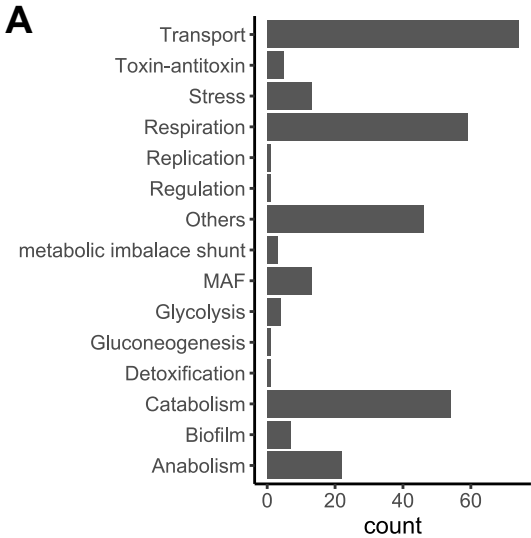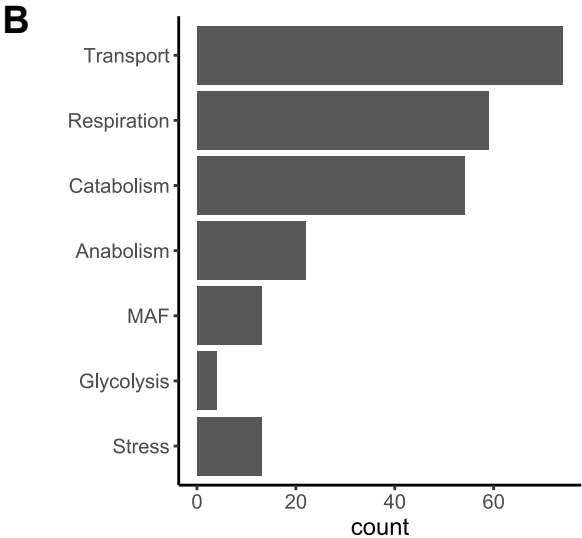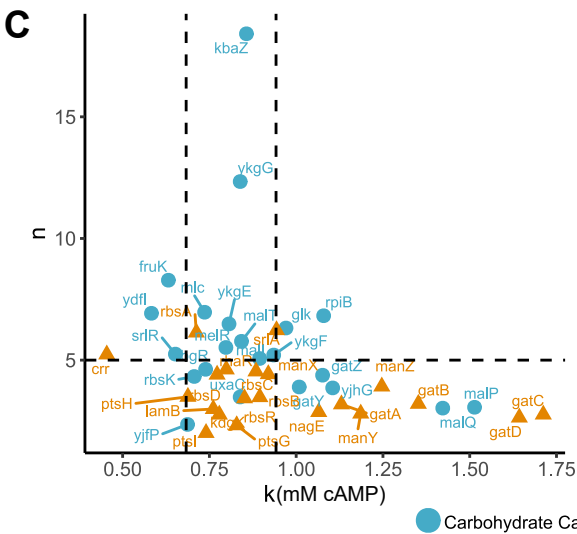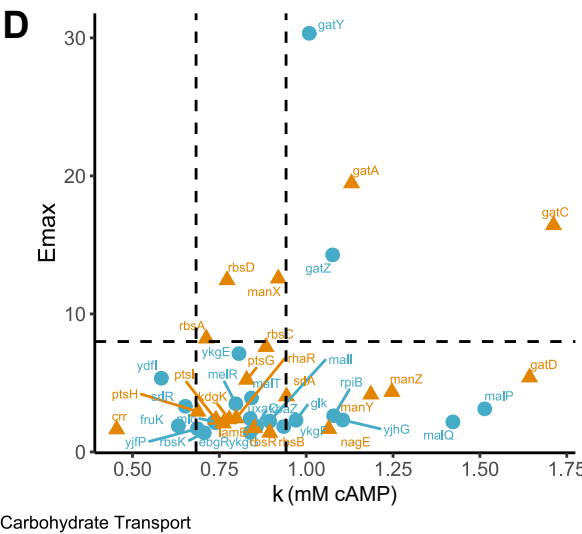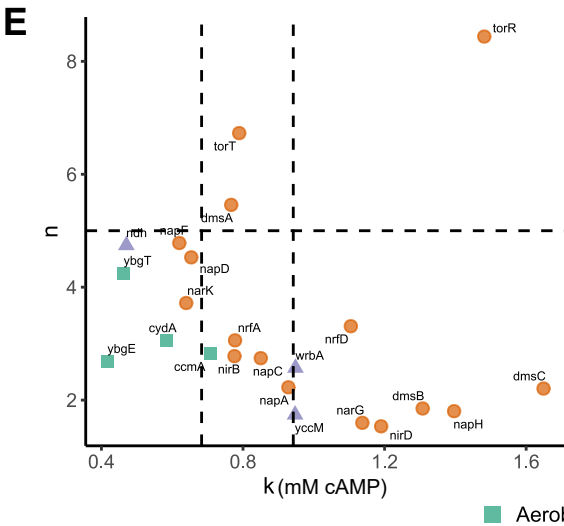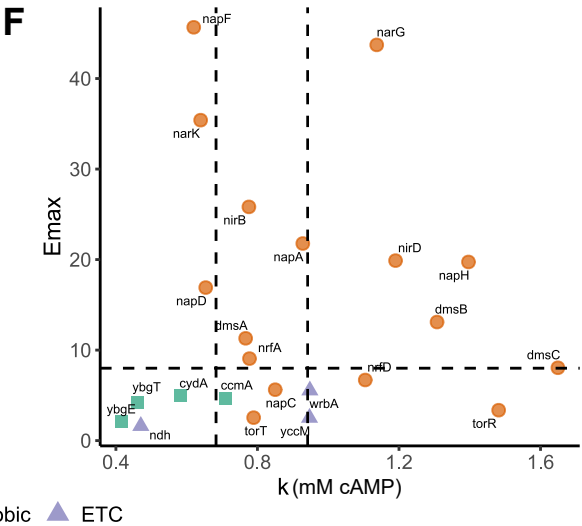

Supplement: FIG S9 [file msystems.00900-22-s0010.pdf]
