# Supplementary material for: Understanding the Genome-Wide Transcription Response To Various cAMP Levels in Bacteria Using Phenomenological Models
Source: mSystems. 2022 Nov 21;7(6):e00900-22. doi: 10.1128/msystems.00900-22 (PMC9765429; doi:10.1128/msystems.00900-22)
Supplement: FIG S2 [file msystems.00900-22-s0003.pdf]

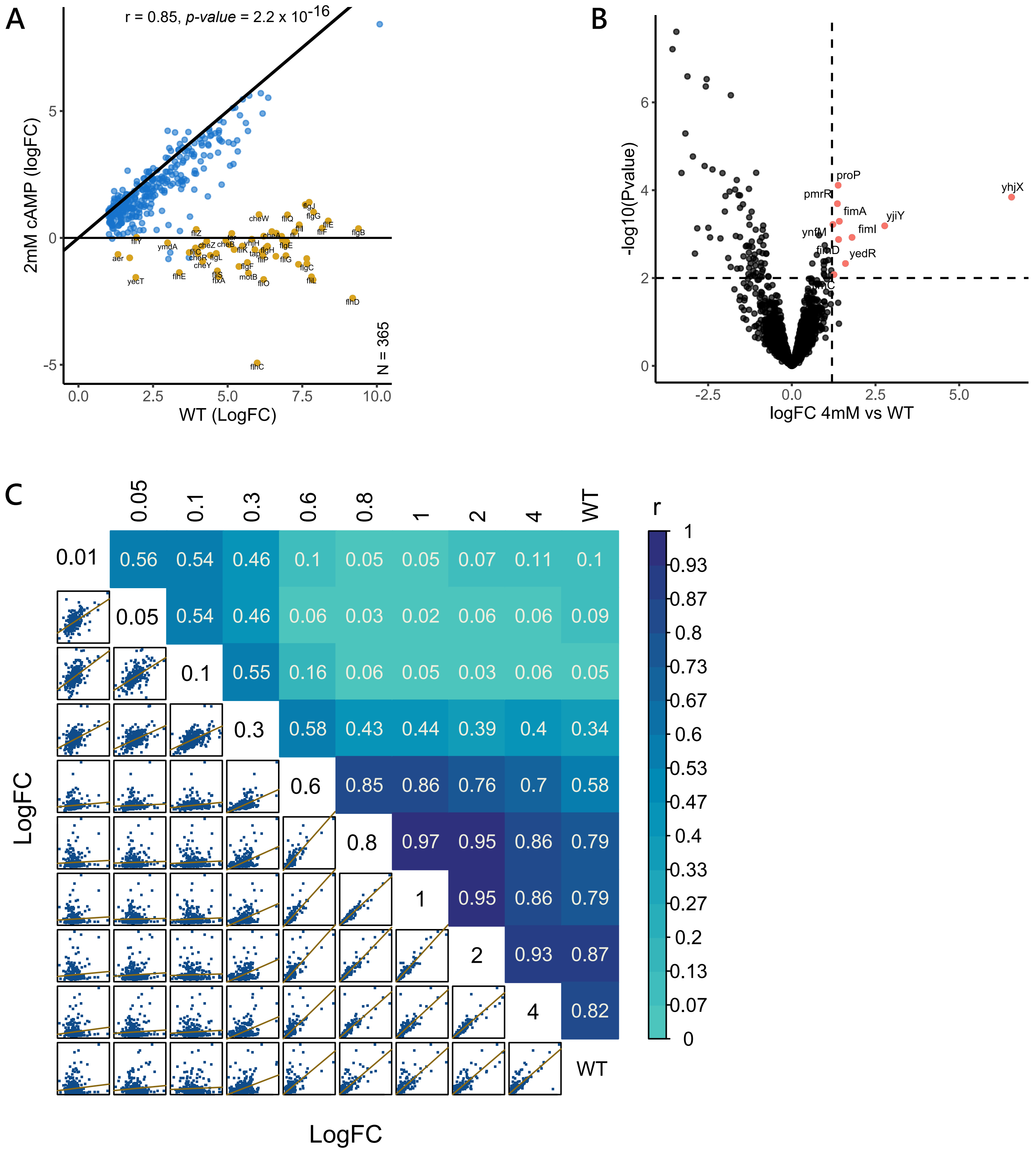

A
r = 0.85, p-value = 2.2 x 10^-16
2mM cAMP (logFC)
WT (LogFC)
N = 365
flhD
flhC
B
-log10(Pvalue)
logFC 4mM vs WT
yhjX
proP
pmrR
fimA
yjiY
fimI
ynfM
fimD
yedR
C
0.01
0.05
0.1
0.3
0.6
0.8
1
2
4
WT
0.56 0.54 0.46 0.1 0.05 0.05 0.07 0.11 0.1
0.54 0.46 0.06 0.03 0.02 0.06 0.06 0.09
0.55 0.16 0.06 0.05 0.03 0.06 0.05
0.58 0.43 0.44 0.39 0.4 0.34
0.85 0.86 0.76 0.7 0.58
0.97 0.95 0.86 0.79
0.95 0.86 0.79
0.93 0.87
0.82
r
LogFC
LogFC
